# Immune Responses to Respiratory Syncytial Virus Vaccines: Advances and Challenges

**DOI:** 10.3390/microorganisms12112305

**Published:** 2024-11-13

**Authors:** Gabriela Souza da Silva, Sofia Giacomet Borges, Bruna Bastos Pozzebon, Ana Paula Duarte de Souza

**Affiliations:** Laboratory of Clinical and Experimental Immunology, Infant Center, School of Health Science, Pontifical Catholic University of Rio Grande do Sul (PUCRS), Porto Alegre 90619-900, Brazil

**Keywords:** vaccine, Respiratory Syncytial Virus, immune response

## Abstract

Respiratory Syncytial Virus (RSV) is a leading cause of acute respiratory infections, particularly in children and the elderly. This virus primarily infects ciliated epithelial cells and activates alveolar macrophages and dendritic cells, triggering an innate antiviral response that releases pro-inflammatory cytokines. However, immunity generated by infection is limited, often leading to reinfection throughout life. This review focuses on the immune response elicited by newly developed and approved vaccines against RSV. A comprehensive search of clinical studies on RSV vaccine candidates conducted between 2013 and 2024 was performed. There are three primary target groups for RSV vaccines: pediatric populations, infants through maternal immunization, and the elderly. Different vaccine approaches address these groups, including subunit, live attenuated or chimeric, vector-based, and mRNA vaccines. To date, subunit RSV vaccines and the mRNA vaccine have been approved using the pre-fusion conformation of the F protein, which has been shown to induce strong immune responses. Nevertheless, several other vaccine candidates face challenges, such as modest increases in antibody production, highlighting the need for further research. Despite the success of the approved vaccines for adults older than 60 years and pregnant women, there remains a critical need for vaccines that can protect children older than six months, who are still highly vulnerable to RSV infections.

## 1. Introduction

Human Respiratory Syncytial Virus (RSV) is one of the most prevalent agents of acute respiratory infection in children worldwide and one of the leading causes of pediatric hospitalization [1]. By the age of 2, most children have been infected at least once by the virus; however, the peak incidence occurs between 2 and 3 months of age [2]. Protection against reinfection by RSV is short-lived, so reinfections are common throughout life. After RSV infection, clinical manifestations can range from mild upper respiratory tract illnesses to severe and potentially fatal lower respiratory tract conditions. Bronchiolitis is the most common lower respiratory tract infection condition, followed by pneumonia. Infants and the elderly have a higher risk of disease complications.

RSV was first isolated in 1956, from material collected from a chimpanzee with a runny nose [3], and it was identified in 1961 for the first time in infants and children during a bronchiolitis epidemic [4]. RSV belongs to the *Pneumovirus* family, of the genus *Orthopneumovirus* [5], and it is an enveloped RNA virus, single-stranded, with a non-segmented negative-sense genome. There are three proteins in the viral envelope, the Fusion F glycoprotein responsible for the virus entering the cells, the attachment G glycoprotein, and the small hydrophobic protein (SH), a surface transmembrane protein. The virus also contains five other structural proteins, the large L protein, nucleocapsid (N), phosphoprotein (P), matrix (M), and M2-1, in addition to two non-structural proteins, NS1 and NS2 [6]. Two distinct RSV groups, A and B, are classified based on epitope differences in the G protein.

The innate immune response plays a crucial role in RSV infection. This virus infects ciliated epithelial cells and activates alveolar macrophages and dendritic cells, triggering an innate antiviral response. These cells express pattern recognition receptors (PRRs), primarily Toll-like receptors (TLRs), RIG-I-like receptors, and NOD2-like receptors. The interaction of RSV with TLRs activates the transcription factor signaling pathway (NF-kB), promoting the production of pro-inflammatory cytokines and increasing the expression of TLRs by epithelial cells and monocytes [7]. Plasmacytoid dendritic cells (pDCs) increase the expression of TLR7, which, when activated, results in the production of pro-inflammatory cytokines and Type I Interferon (IFN-I) [8]. The intracellular receptor RIG-I recognizes triphosphorylated viral RNA, interacts with the mitochondrial antiviral signaling protein (MAVS), and induces the production of pro-inflammatory cytokines and IFN-I through the NF-kB pathway. The single-stranded viral genome is also recognized by the cytoplasmic receptor NOD2, promoting the production of IFN-I [7]. The interaction of NOD-like receptors with the RSV SH protein induces the assembly of the inflammasome in lung epithelial cells, generating the cytokine IL-1β. Such mechanisms result in the production of pro-inflammatory cytokines, chemokines, and adhesion molecules and the activation of NK cells, granulocytes, monocytes, macrophages, and dendritic cells [9,10]. The adaptive immune response is activated, consisting of humoral immunity (the development of short-lived protective antibodies) and cell-mediated immunity (important in modulating RSV pathogenesis) [11]. During acute RSV infection, there is an increased expression of Fas and TRAIL receptors on circulating T cells, along with an increase in systemic concentrations of soluble Fas ligand and caspase-1, leading to transient lymphopenia [12]. T-cell lymphopenia is more evident in younger individuals, in severe cases of the disease and in patients requiring ventilatory support [12,13].

The antibody response is mainly directed against viral surface glycoproteins, such as the G and F proteins [14]. Serum-neutralizing antibodies and mucosal IgA and IgG directed against glycoprotein G are specific to the RSV group [15]. In contrast, antibodies against glycoprotein F exhibit cross-reactivity between different RSV groups A and B. IgA plays a key role in protecting the upper respiratory tract both in the short-lived response after primary infection and in the long-lasting response in reinfection episodes [16]. During primary RSV infection in young infants, maternal IgG antibodies against RSV can inhibit the generation of an IgA antibody response directed at RSV at the site of respiratory disease [17,18]. Serum IgG offers considerable but limited protection against RSV infections; the short lifespan of IgG and the immune evasion strategies of the virus explain the high incidence of reinfections after primary infection [19,20].

There are three targets for RSV vaccines: the pediatric population, infants through maternal immunization, and the elderly [21]. Different vaccine approaches can therefore be used: subunit vaccines, live attenuated or chimeric vaccines, vector-based vaccines, and mRNA vaccines. During the development a vaccine against RSV, there have been different obstacles, such as concerns about increased respiratory disease after vaccination, which occurred with the formalin-inactivated vaccine in the 1960s, leading to delays in developing new vaccines. Other challenges are finding an absolute correlation of protection against the virus since there is a lack of knowledge about the immunological mechanisms of virus protection and natural reinfection can occur frequently, and there are inadequacies in the design and implementation of clinical studies [22]. Considering all this information, we decided to review the approved RSV vaccines and those under development in the current scenario, mainly focusing on the immune response.

## 2. Materials and Methods

A comprehensive search of five databases—PubMed/MEDLINE, EMBASE, Li-lacs/BSV, Scielo, and Science.gov—was performed between 2023 and 2024. The search strategy used a combination of the terms: “vaccine”, “respiratory syncytial virus” and “clinical trials”. Articles were included based on two eligibility criteria: a publication date within the 2013–2024 timeframe and their designation as clinical studies. A total of seventy-three articles were identified, of which eleven were excluded for not meeting the eligibility criteria.

## 3. Results and Discussion

**Figure 1:**
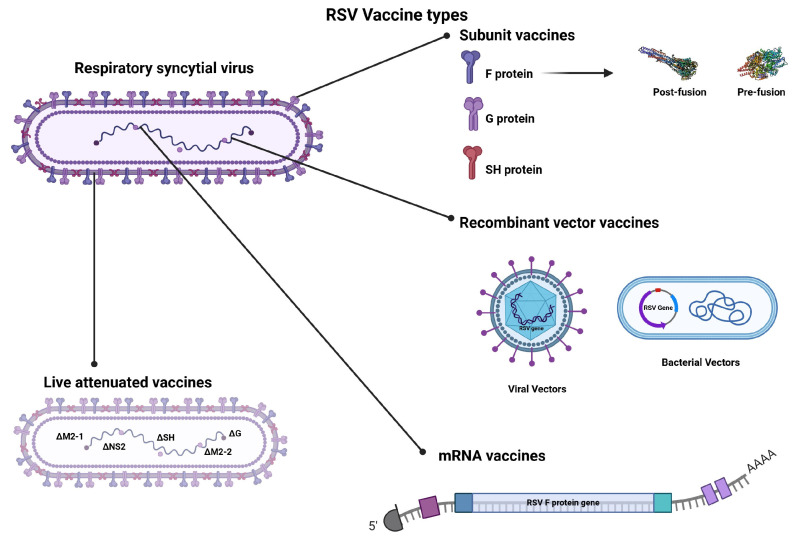
RSV vaccine types in clinical trials and approved for use. There are different RSV vaccine types: subunit vaccines, recombinant vector vaccines, live attenuated vaccines, and mRNA vaccines. Three RSV proteins, F, G, and SH, were tested in the subunit vaccine approach. The F protein-based vaccines used two different F protein conformations, post-fusion and pre-fusion. The RSV vaccines approved for use in the elderly and pregnant are subunit and mRNA vaccines based on the pre-fusion conformation.

Sixty-two articles on clinical studies of RSV vaccines were included in the final selection. Among these, thirty-two focused on subunit vaccine studies, seventeen on recombinant vector vaccines, nine on live attenuated virus vaccines, and four on mRNA vaccines. A summary of the types of RSV vaccines studied is presented in Figure 1.

### 3.1. Subunit Vaccines

Subunit vaccines consist of subcomponents, usually proteins or polysaccharides, instead of the entire virus. They are often developed using recombinant DNA technology. The advantages of this type of vaccine are the reduced risk of side effects because these vaccines do not contain live pathogens, specificity since they are developed using the most immunogenic part of the virus, and stability. However, subunit vaccines induce a weak immune response compared to live vaccines, and normally, booster doses or adjuvants are required. The immunogenicity of subunit vaccines can be enhanced through aggregates, virus-like particles, antigens encapsulated by lipids, or even antigen-coated spheres [23].

#### 3.1.1. Post-Fusion F Vaccines

The RSV sF vaccine, funded by MedImmune, is a vaccine against RSV that contains the virus’s soluble fusion protein (sF) in its post-fusion form. In 2016, a phase Ia placebo-controlled study was conducted on healthy volunteers aged 60 or older at three sites in the United States. The study assessed the safety and tolerability of a single ascending dose of this vaccine, both alone and in the presence of the adjuvant glucopyranosyl lipid A (GLA-SE), formulated in a stable squalene-based oil-in-water emulsion. This adjuvant is a synthetic analog of monophosphoryl lipid A, a Toll-like receptor-4 agonist that stimulates immune responses, leading to a type 1 T-helper response to the vaccine. Regarding immunogenicity, all dosage levels elicited immune responses superior to those of placebo recipients. Immune responses induced by the vaccines were enhanced in a dose-dependent manner, particularly with the use of the GLA-SE adjuvant [24]. The details of immunogenicity are described in Table 1. In 2017, a second trial of the same RSV sF vaccine was published [25]. It was a phase Ib study in healthy volunteers aged 60 or older to provide safety and immunogenicity data for use and selection of a dose in a subsequent phase II efficacy study. The results confirm the immunogenicity (Table 1) and safety of the vaccine and the adjuvant, providing a basis for dose selection in a phase II study [25]. In the same year, 2017, a new trial was published regarding the RSV sF vaccine, now called MEDI7510 [26]. It was a phase IIb study conducted on healthy volunteers aged 60 years and older in the United States, Canada, Eastern Europe, Chile, and South Africa. The study evaluated the safety, immunogenicity, and efficacy of 120 µg vaccine with 5 µg of the GLA-SE adjuvant. The most reported local adverse events were pain and tenderness at the injection site. For systemic adverse events, vaccine recipients reported more fatigue and muscle pain compared to placebo recipients. The geometric mean levels of anti-F IgG increased approximately 1.5 times from baseline on day 29 post vaccination. The geometric mean of RSV A microneutralizing antibody titers was approximately twice the baseline values (Table 1). Regarding efficacy, 25.8% of individuals who received the vaccine had an episode of illness compared to 28.6% of individuals in the placebo group. Although immunogenic, this vaccine was ineffective in preventing RSV disease in older adults. The authors concluded that this failure was most likely due to the post-fusion conformation of the virus’s F protein not generating appropriate neutralizing antibodies for disease prevention [26].

Another post-fusion vaccine tested was the RSV F vaccine, funded by Novavax Inc. [27]. This vaccine contains RSV F proteins produced in Sf9 insect cells coated with nanoparticles and using aluminum phosphate as an adjuvant (AdjuPhos). The aluminum phosphate adjuvant is prepared by precipitating aluminum ions under alkaline conditions in the presence of phosphate [27]. This adjuvant induces the production of IL-1β and IL-18 by dendritic cells and peripheral blood mononuclear cells in a caspase-1-dependent manner and enhances antigen presentation and T-cell activation [28]. In a phase I study, in healthy volunteers aged 18 to 49, this vaccine was evaluated for dose escalation, safety, reactogenicity, and immunogenicity against RSV. All vaccine formulations administered were well tolerated, with no significant dose-related toxicity observed. A dose–response relationship was observed in the groups containing the adjuvant to antibody levels (Table 1). Following this study, a phase II, placebo-controlled study was conducted at four sites in the United States in healthy women aged 18 to 35 years, who were not pregnant and not lactating with the same vaccine preparation [29]. The addition of the aluminum adjuvant positively affected the immune response, resulting in a 1.4-fold increase in antibody levels on day 28 and antibody levels 1.7 times higher on day 56 compared to vaccine recipients without the adjuvant. Considering all these factors, it was possible to confirm the benefit of a higher antigen dose, the enhancement of immunity with aluminum phosphate as an adjuvant, and the potential for developing a single-dose vaccine [29] (Table 1). In 2017, another study was published on the RSV F vaccine in healthy, non-pregnant, and non-lactating women of reproductive age [30]. This phase II, placebo-controlled study was conducted in 10 centers in the United States to evaluate the safety and immunogenicity of six formulations, assessing the 120 μg dose of the RSV F vaccine in a single dose with varying amounts of aluminum phosphate adjuvant compared to two 60 μg doses with the adjuvant also in different dosages to select the best candidate. All the formulations were well tolerated. The dose of 120 µg responses showed higher peaks of anti-F IgG antibodies 14 days post vaccination compared to the double-dose 60 µg vaccine, regardless of the adjuvant level. However, in the 0.4 and 0.2 mg adjuvant doses, the peak response lasted longer, extending for four to five weeks (Table 1) [30]. In the same year, another study was published, testing the RSV F vaccine in healthy volunteers aged 60 years or older; this was a phase I, placebo-controlled study that evaluated the safety and immunogenicity of a single immunization with a 60 or 90 μg dose of the vaccine with or without aluminum phosphate, conducted in four clinical centers in the United States [31]. The 90 μg dose produced stronger responses with or without the adjuvant compared to the 60 μg dose, and there was a significant increase in adjuvanted formulations on day 56 (Table 1) [31]. The next study on the RSV F vaccine by Novavax was published in 2019 [32]. It was a phase II, placebo-controlled study conducted in healthy pregnant women aged 18 to 40 years, between 33 and 35 weeks of gestation, across eight centers in the United States. The study evaluated the safety and immunogenicity of the vaccine and the placental transfer and decay kinetics of specific RSV antibodies in newborns. The vaccine was well tolerated, with no significant differences observed between vaccine and placebo recipients in reporting unsolicited adverse events, medical consultations for adverse events, or severe adverse events. The immunization was also safe for newborns, with no significant differences in average gestational age at birth, birth weight, or APGAR scores. Newborns of vaccinated women had significantly higher antibody levels in umbilical cord blood compared to newborns whose mothers received a placebo, with the half-lives of RSV-specific antibodies in infants approximated to be 40 days (Table 1). In 2020, the next study on the RSV F vaccine by Novavax was published [33]. This was a phase III, placebo-controlled trial conducted in healthy pregnant women aged 18 to 40 years, with gestation between 28 weeks and 0 days and 36 weeks and 0 days. The study was conducted across 87 locations in countries including Argentina, Australia, Chile, Bangladesh, Mexico, New Zealand, the Philippines, South Africa, Spain, the United Kingdom, and the United States. The results showed that the vaccine presented an acceptable safety profile. Fourteen days post vaccination, the geometric mean concentration of palivizumab-competitive antibodies was approximately 12.4 times higher than baseline. The antibody half-life in the infants in the vaccine group was 49.1 for palivizumab-competitive antibodies and 38.3 for anti-F IgG. The primary efficacy of the vaccine was 39%, which did not meet the pre-specified criterion [33].

Other studies tested an RSV F in a post-fusion conformation vaccine funded by GlaxoSmithKline Biologicals SA. This vaccine was tested in a phase I, placebo-controlled study in non-pregnant women and healthy men aged 18 to 45 years, aimed to evaluate the safety and immunogenicity of different doses and formulations of this experimental vaccine [34]. The vaccine was tested without adjuvants and with the following adjuvants: aluminum hydroxide (Al) and MF59. MF59 is an adjuvant based on a squalene and surfactant emulsion designed to induce a more robust, broader, and longer-lasting immune response [35]. The highest geometric mean titers of anti-RSV neutralizing antibodies were observed with the vaccine containing the Al adjuvant (Table 1). Additionally, it was observed that the second dose did not demonstrate any booster effect. In this study, the author mentions that recent studies have shown that the pre-fusion conformation of the RSV F protein has greater neutralizing activity than antibodies targeting the F protein in its post-fusion conformation [34].

**Table 1:** Results of the RSV subunit vaccines clinical trials using post-F conformation.

Study Design	Study Population	Year of Publication	Vaccine	Dose	Data Analyis	Immunogenicity (anti-F igG Response)	Microneutralization Titers		Competition with Palivizumab	Reference
Phase Ia	n = 144 (24 per cohort)	2016	RSV sF post-fusion	80 μg + GLA-SE	29 days	More than 1000 unit/mL	VSR A	VSR B	More than 100 µg/mL (mean)	[24]
	n = 144 (24 per cohort)						Between 640 and 2560 GMT			
Phase Ib	n = 264	2017	RSV sF post-fusion	120 μg + 1 μg GLA-SE	29 days	More than 1000 unit/mL (mean)	VSR A	VSR B	Less than 100 µg/mL (mean)	[25]
	Healhy adults aged 60						Between 1024 and 2048 GMT			
Phase Ib	n = 1894	2017	RSF sF post-fusion	120 μg + 1 μg GLA-SE	29 days	More than 1000 unit/mL (mean)	VSR A	VSR B	Less than 100 µg/mL (mean)	[26]
	Healthy volunteers aged 60 years and older						Between 1024 and 2048 GMT			
Phase I	n = 150	2013	RSV F post-fusion	60 μg	30 days	5652 GMT	VSR A	VSR B	[30]	[27]
	Healthy adults aged 18 to 49 year						985 GMT	782 GMT		
Phase II	n = 300	2015	RSV F post-fusion	90 µg + Al	28 days	1 dose: 11,109 GMT 2 doses: 9577 GMT	VSR A	VSR B	1 dose: 275 GMC 2 doses: 252 GMC	[29]
	Women (18–35 years)						1 dose: 1482 GMT 2 doses: 1226 GMT	1 dose: 362 GMT 2 doses: 200 GMT		
Phase II	n = 761	2017	RSV F post-fusion	120 μg + 0.2 Al	28 days	10,000 GMEU	VSR A	VSR B	Less than 400 GMC	[30]
	Women (18–35 years)						Less than 800 GMT	Between 400 and 800 GMT		
Phase I	n = 220	2017	RSV F post-fusion	90 µg	28 days	More than 6000 GMEU	VSR A	VSR B	More than 160 GMC	[31]
	Healthy volunteers aged 60 years and older						Increase of more than 1.4 times in GMT parameters compared to baseline levels	Increase of more than 1.3 times in GMT parameters compared to baseline levels		
Phase II	n = 50	2019	RSV F post-fusion	120 µg + 0.4 Al	On the day of delivery in newborns	7243.6 GMEU	VSR A	VSR B	182 GMC	[32]
	Healthy pregnant women (18–40 years)						759.1 GMT	480.7 GMT		
Phase I	n = 288	2019	RSV F post-fusion	135 µg + Al	29 days	13,514 GMT	VSR A			[34]
	Non-pregnant women and men aged 18–45 years						More than 1500 GMT			

GLA-SE: Toll-like receptor 4 (TLR-4) agonist adjuvant, glucopyranosyl lipid A (GLA), in a squalene-based stable emulsion (SE); GMT: geometric mean antibody titer; Al: adjuvant aluminum phosphate; GMC: geometric mean concentration; GMEU: geometric mean ELISA unit.

#### 3.1.2. Pre-Fusion F Vaccines

The RSV-PreF vaccine was developed in the pre-fusion conformation of the RSV F glycoprotein as a specific epitope in this conformation, called site ø (zero), is considered one of the main targets of neutralizing antibodies [36]. One vaccine produced using the pre-fusion conformation of F protein RSV subtype A was DS-Cav1, which retains multiple antigenic sites sensitive to neutralization at the apex of the trimer. This vaccine was tested in a phase I trial in healthy adults aged 18 to 50 recruited in the Washington metropolitan area. The study aimed to evaluate this new vaccine’s dose, safety, tolerability, and immunogenicity, alone or with the alum adjuvant (aluminum hydroxide with Alhydrogel 2%) (Table 2). RSV A neutralizing activity decreased between 4 and 44 weeks after vaccination but remained 4.5 times above baseline activity in the group that received the 500 mcg dose. After 44 weeks of vaccination, there was no significant difference in neutralizing activity between participants who received one or two doses of the vaccine. The alum adjuvant had no considerable impact on immunogenicity [37].

The SynGEM vaccine is another subunit vaccine containing the F protein in its pre-fusion conformation of RSV, linked to a bacterial peptidoglycan-like particle (BLP) derived from the Lactococcus lactis bacterium [38]. It was developed for intranasal administration. This vaccine was tested in a phase I, placebo-controlled study in healthy adults aged 19 to 49 years. The study assessed safety, tolerability, and immunogenicity (Table 2). The vaccine-induced antibodies were predominantly non-neutralizing. This could have been due to the limited overall size of antibody responses or the poor in vivo presentation of the F protein, resulting in low neutralizing antibody generation. However, the vaccine induced mucosal RSV-specific IgA in most individuals, particularly in those with low pre-existing titers of this antibody [38].

The first phase I trial that tested the vaccine produced in the pre-fusion conformation (RSV-PreF), funded by GlaxoSmithKline Biologicals SA, was a placebo-controlled study in healthy men aged 18 to 44 years, conducted at three sites in Canada. The study aimed to evaluate the safety, reactogenicity, and immunogenicity of a single dose of six vaccine formulations, with or without an aluminum hydroxide (alum) adjuvant. The vaccine was considered safe and had acceptable reactogenicity. All vaccine recipient groups showed an increase in neutralizing antibodies (Table 2). As a result, the authors selected the higher doses for further studies [39]. A report of the results of two trials of this vaccine was published in 2018 [40]. The combined tetanus toxoid–diphtheria and toxoid–acellular pertussis (Tdap) vaccine was used as a control vaccine. The phase II trial, named RSV F-020, was a controlled study conducted at multiple sites in Australia, the United States, the Czech Republic, and Germany, evaluating the reactogenicity and immunogenicity of three different formulations of the RSV-PreF vaccine with or without the aluminum hydroxide adjuvant in non-pregnant women of reproductive age, aged 18 to 45 years. Following the results of this study, the 60 µg vaccine was selected for an additional phase II trial, named RSV F-024, also evaluating safety in non-pregnant women of reproductive age (18 to 45 years), conducted at a single center in Belgium. This trial used vaccines without an adjuvant and with an aluminum hydroxide adjuvant and the Tdap vaccine for adults with an aluminum adjuvant as the control vaccine. Of the 14 pregnancies developed in 13 women during this trial, there were two induced abortions and two spontaneous abortions, which were deemed to have no reasonable possibility of being caused by the experimental vaccine. The vaccine formulation that reached the highest values of RSV A and RSV B neutralizing antibodies was the 60 µg dose without an adjuvant (Table 2) [40]. In 2019, a new study of the same RSV-PreF vaccine, phase II, placebo-controlled trial, was conducted in non-pregnant women of reproductive age at eight centers in Belgium, Estonia, France, and Germany [41]. The doses of 30 µg, 60 µg, and 120 µg were used to evaluate safety, reactogenicity, and immunogenicity. There was no significant difference when comparing the 120 µg dose to the 60 µg dose (Table 2) [41]. Although there were no safety concerns with this vaccine, its development was discontinued due to the instability of the RSV-PreF antigen during manufacturing.

In 2021, the GSK group published a new trial, this time with a new vaccine called RSVPreF3, a modified version of the F protein stabilized in its pre-fusion conformation through the introduction of cysteine residues [42]. This was a phase I/II, placebo-controlled study in healthy, non-pregnant women aged 18 to 45 years, conducted at two centers in the United States, four centers in Finland, and five centers in Germany. The study evaluated the safety, reactogenicity, and immunogenicity of this vaccine. The 60 µg and 120 µg doses were found to be the most immunogenic, but with the higher dose, the levels of antibodies decreased over time but remained 7.6 times higher on day 91 (Table 2) [42]. In 2022, the results of a phase I/II, placebo-controlled study using RSVPreF3 in adults aged 18 to 40 years and in elderly people aged 60 to 80 years, conducted at various centers in the United States and Belgium, were published [43]. The safety, reactogenicity, and immunogenicity of the vaccine with or without an AS01-based adjuvant (AS01 E and AS01 B) were evaluated. AS01 is a liposome-based adjuvant capable of inducing robust CD4 T helper cell responses and rapid and longer-lasting humoral and cellular responses when combined with the antigen. The AS01 E adjuvant is composed of 25 µg of 3-O-desacyl-4′-monophosphoryl lipid A [MPL], 25 µg of Quillaja saponaria Molina, fraction 21 [QS-21], and liposome, while AS01 B is composed of 50 µg of MPL, 50 µg of QS-21, and liposome. This adjuvant was tested only in the elderly group. The highest responses were observed on day 31, with no additional effect from administering a second vaccine dose, and the best benefit was seen in formulations containing 120 µg (Table 2). Based on these results, the chosen vaccine for further studies was the 120-AS01 E formulation in a single dose [43]. In January 2023, another study of the same RSVPreF3 vaccine combined with the AS01 B adjuvant was published. This was a phase I, placebo-controlled study in adults aged 60 to 80 years old, carried out at a center in Japan [44]. The study aimed to assess safety, reactogenicity, and vaccine immunogenicity (Table 2). The authors concluded that the 120 μg vaccine combined with the AS01 E adjuvant system demonstrated lower reactogenicity and no increase in the immunogenicity with the second dose [44]. In the same year, the results of a phase III study were published testing the RSVPreF3 vaccine [45]. It was a placebo-controlled study in adults aged 60 years or older, conducted in 17 countries across Africa, Asia, Australia, Europe, and North America. The study evaluated the efficacy of a single 120 μg dose of the vaccine with the AS01 E adjuvant system in preventing lower respiratory tract disease caused by RSV during the RSV season. It also evaluated reactogenicity, safety, and immunogenicity (Table 2) [45]. No imbalances were observed between the vaccine and placebo groups in the overall incidence of serious adverse events. The primary efficacy was 82.6%. The presented data demonstrated that this vaccine has an acceptable safety profile and prevents acute respiratory infection, lower respiratory tract disease, and severe lower respiratory tract disease, regardless of the RSV subtype [45]. This vaccine was approved on May 23, 2023, for use in individuals aged 60 years or older under the trade name AREXVY by the United States Federal Drug Administration (FDA) [46].

In 2024, a study investigated the efficacy and safety of this vaccine across two RSV seasons with a single dose and a first dose followed by revaccination after one year with the AS01-adjuvanted RSVPreF3 (AREXVY vaccine). All vaccine dosages demonstrated an acceptable safety profile. The efficacy of a single dose of the vaccine in preventing RSV-associated lower respiratory tract disease over two seasons was 67.2%. The efficacy over two seasons for severe RSV-associated lower respiratory tract disease was 78.8%, and for RSV-associated acute respiratory infection, it was 52.7%. Sustained efficacy was observed over both seasons, but estimates tended to decrease. The vaccine’s efficacy with a revaccination regimen after one year over two seasons was 67.1% against RSV-associated lower respiratory tract disease, and there was an efficacy of 78.8% and 60.3% for RSV-associated acute respiratory infection. These data demonstrate that revaccination after one year of the first dose is well tolerated but does not provide additional efficacy benefits [47]. Also, the persistence of the immune response was evaluated in an ongoing phase III study after three years of RSVPreF3 OA immunization. The neutralization antibody titers declined over time, but they remained above pre-dose 1 levels for at least one year [48].

The RSVPreF3 was also tested in healthy pregnant women aged 18 to 40 years [49]. A phase II trial of the same RSVPreF3 vaccine was conducted using 60 µg and 120 µg doses without an adjuvant. A placebo-controlled clinical trial evaluated its safety, reactogenicity, and immunogenicity in healthy pregnant women aged 18 to 40 years, conducted in Australia, Canada, Finland, France, New Zealand, Panama, South Africa, Spain, and the United States [49]. Serious adverse events were reported by 47.1% and 53.3% of mothers, with the most common event being fetal distress syndrome, reported more frequently (10.7%) in the 120 µg dose vaccine group. After 6 weeks of postpartum, different congenital anomalies were reported in the infants. In the 60 µg vaccine group, there were two cases. In the 120 µg vaccine group, there were four cases, and in the placebo group, there was only one case. In infants, the geometric means of IgG antibody placental transfer rates reached 1.90 in the group that received the 120 µg dose (Table 2) [49]. On 18 February 2022, the company GKS announced that it had voluntarily halted enrollment and vaccination in the phase III trial of the vaccine in healthy pregnant women aged 18 to 40 years due to recommendations from the Independent Data Monitoring Committee, and based on observations from routine safety assessments, additional analyses are underway to understand better the safety data from these trials [50,51]. The data of this phase III trial was published in 2024. The vaccine’s efficacy in preventing RSV-associated lower respiratory tract disease from birth to six months of age was 65.5%, and for severe RSV-associated lower respiratory tract disease, it was 69.0%. Adverse events of special interest and serious adverse events were similar in both groups, except for preterm birth and neonatal death in infants. After the study was halted, it was found that preterm birth occurred in 6.8% of infants in the vaccine group compared to 4.9% in the placebo group. Among preterm infants, neonatal death occurred in seven infants in the vaccine group and none in the placebo group. The mechanism by which the vaccine might have led to a higher risk of preterm birth compared to the placebo group remains unknown [52].

In a similar rationale using the prefusion conformation, a bivalent prefusion F vaccine (RSVpreF) containing trimeric F glycoproteins from both major RSV subgroups (A and B) was tested to protect older adults directly and protect infants via maternal immunization during pregnancy [53]. This vaccine, funded by Pfizer, was published in a 2022 phase I/II placebo-controlled study in healthy adults aged 18 to 85, conducted at 36 sites in the United States [54]. The study evaluated dose variation and the safety and immunogenicity of three dose variations with or without an aluminum hydroxide adjuvant. The adjuvant did not enhance the neutralizing responses (Table 2). Based on the results obtained in this study, the authors selected the 120 µg dose without the adjuvant for further evaluation as this dosage presented a better safety profile [54]. A second phase I/II trial was also conducted with healthy adults aged 18 to 49 in 40 sites in the USA immunizing with the bivalent vaccine [55]. The results demonstrated that the vaccine is safe and highly immunogenic, and the adjuvant did not influence the increase in response (Table 2) [55]. The addition of the CpG oligodeoxynucleotide adjuvant linked to aluminum hydroxide was evaluated in a phase I/II study conducted in 12 sites in Australia in healthy volunteers aged 65 to 85 years [56]. CpG oligodeoxynucleotide is a Toll-like receptor 9 agonist and its addition may further improve humoral and cellular responses in elderly participants. The safety and immunogenicity of the formulations with and without an adjuvant were also evaluated. The results showed no difference in the immune response between the formulations with adjuvants. The reason for the lack of effect of CpG plus aluminum hydroxide is unknown [56]. The bivalent RSV prefusion F protein-based (RSVpreF) vaccine candidate was also tested in an RSV challenge trial to obtain the proof of concept before phase III, designed as a phase IIa placebo-controlled trial in adults aged 18 to 50 years to assess the effectiveness of the RSV vaccine [57]. The geometric mean titers of neutralizing antibodies compared to the baseline value were 20.5 times for RSV A and 20.3 times for RSV B (Table 2). The vaccine’s efficacy against virus infection, regardless of the presence, absence, or severity of symptoms with a PCR result in at least 2 days, was 75% and its efficacy was 100% for any quantifiable infection confirmed by culture. All these data show that this vaccine protects against symptomatic RSV infection in a viral challenge model [57]. Finally, in 2023, the results were published of the phase III study, placebo-controlled, conducted in adults aged at least 60 years who were healthy or had stable chronic conditions and carried out at 240 sites in Argentina, Canada, Finland, Japan, the Netherlands, South Africa, and the United States [58]. Two serious adverse events were identified, one case of Guillain–Barré syndrome and one case of Miller Fisher syndrome in the group of vaccine recipients, and both had confounding factors that made it difficult to discern their potential relationship with the vaccine. Although both cases occurred in an age group at an increased risk of these syndromes, these adverse events warrant close monitoring if the vaccine is approved and recommended in future studies and with post-marketing surveillance. There was 66.70% efficacy for the primary endpoint of respiratory disease with at least two symptoms and 85.70% efficacy for respiratory disease with at least three symptoms. For the secondary endpoint, there was 62.10% efficacy in episodes of acute respiratory illness with at least one symptom. All the efficacy criteria were met and the RSVpreF vaccine had an acceptable safety profile [58]. This vaccine was approved on 31 May 2023, for use in individuals aged 60 and over under the trade name Abrysvo by the Federal Drug Administration (FDA), a US government agency [59].

Focusing on the protection of the infant population, this bivalent vaccine RSVpreF was tested in a phase IIb, placebo-controlled study in healthy pregnant women between 18 and 49 years of age at 24 to 36 weeks of gestation, carried out in the United States, Chile, Argentina and South Africa [60]. The safety, immunogenicity, transplacental transfer of neutralizing antibodies, and exploratory efficacy of the vaccine in newborns were evaluated. The vaccine dose was 120 or 240 μg of RSVpreF vaccine with or without an aluminum hydroxide adjuvant. Although maternal antibody titers were slightly higher after vaccination in the group containing the aluminum hydroxide adjuvant, it did not show an advantage in infants, in whom the highest antibody titers were seen in the groups without the adjuvant. The vaccine was 84.7% efficient in protecting against virus-associated lower respiratory tract disease with medical care [60]. The phase III study was carried out in 18 countries during the four RSV seasons, and the vaccine dose administered was 120 μg without adjuvant in healthy pregnant women up to 49 years of age at 24 to 36 weeks’ gestation. As a result, the RSVpreF vaccine has shown a promising safety and efficacy profile against severe respiratory tract disease associated with RSV and hospitalization in newborns up to 180 days after birth [61]. This vaccine was approved on 21 August 2023 for use for the first time in pregnant women to prevent RSV in newborns. It was approved under the trade name Abrysvo by the Federal Drug Administration (FDA), a US government agency [62]. In 2024, a subset analysis of the phase III study in Japan was published, confirming the efficacy of the vaccine with no safety concerns [63].

The effectiveness of the immunization with the subunit vaccine against the hospitalization of adults 60 years and older was published in 2024, showing that RSV vaccination was associated with a reduced likelihood of hospitalization with RSV compared to unvaccinated individuals. The study highlights as a limitation the low number of vaccine uptake in the first season [64]

Recently, a meta-analysis has been published evaluating the immunogenicity of subunit RSV vaccines [65]. They found that subunit vaccines significantly augment neutralizing antibody titers among participants, yielding a standardized mean difference of 2.89. The meta-analyzed results showed that these vaccines demonstrate high effectiveness, good safety profiles, and strong immune responses [65]. 

**Table 2:** Results of the RSV subunit vaccine clinical trials using pre-F conformation.

Study Design	Study Population	Year of Publication	Vaccine	Dose	Data Analyis	Immunogenicity (Anti-F igG Response)	Microneutralization Titers		Competition with Palivizumab	Reference
Phase I	n = 244	2021	DS-Cav1 pre-fusion	500 mcg	28 days		VSR A	VSR B		[37]
	Healthy adults aged 18–50 years						7495 GMT	9281 GMT		
Phase I	n = 128	2016	RSV-PreF pre-fusion	30 µg + Alum	30 days	12/14–85.7% (proportion of subjects with response)	VSR A	VSR B	More than 128 GMC	[39]
	18–44-year-old healthy men						More than 1024 and less than 4096 GMT	More than 1024 and less than 4096 GMT		
Phase II	n = 500 (RSV F-020) n = 100 (RSV F-024)	2018	RSV-PreF pre-fusion	60 µg + Al	30 days	69,653.9 GMT	VSR A	VSR B	98.6 GMT/C (geometric mean titer/antibody concentration)	[40]
	Healthy non-pregnant women 18–45 years of age						1371.5 GMT/C			
Phase II	n = 400	2019	RSV-PreF pre-fusion	120 µg	30 days		VSR A	VSR B	More than 64 and less than 128 GMC	[41]
	Healthy non-pregnant women aged 18–45 years						More than 1024 and less than 2048 GMT	More than 1024 and less than 2048 GMT		
Phase I/II	n = 502	2021	RSVPreF3 pre-fusion	120 µg	31 days	89,760.38 GMC	VSR A	VSR B		[42]
	Healthy non-pregnant women 18–45 years of age						7932.35 GMT			
Phase I/II	n = 48 young adults n = 1005 older adults	2022	RSVPreF3 pre-fusion	120 µg + AS01E	31 days	Almost 100,000 CMG	VSR A	VSR B		[43]
	Young adults (18–40 years) and older adults (60–80 years)						Around 10,000 GMT	More than 10,000 GMT		
Phase I	n = 400	2023	RSVPreF3 pre-fusion	120 µg + AS01B	31 days	85,282.4 GMC	VSR A	VSR B		[44]
	Japanese adults aged 60–80 years						5315.0 GMT	9349.1 GMT		
Phase II	n = 213	2023	RSVPreF3 pre-fusion	120 µg	31 days	105,138 GMC	VSR A	VSR B		[47]
	Healthy pregnant women 18–45 years old						10,781 GMT	15,849 GMT		
Phase I/II	n = 617	2022	RSVpreF pre-fusion	240 μg (cohort 18–49 years)	30 days		VSR A	VSR B		[51]
	Adults 18–85 years old (cohort: 18–49 and 65–85 years old)						More than 10,000 and less than 100,000 GMT	More than 10,000 and less than 100,000 GMT		
Phase I/II	n = 618	2022	RSVpreF pre-fusion	240 μg	1 month		VSR A	VSR B		[52]
	Adults 18–85 years old (cohort: 18–49 and 50–85 years old)						More than 10,000 and less than 100,000 GMT	More than 10,000 and less than 100,000 GMT		
Phase I/II	n = 317	2021	RSVpreF pre-fusion	240 μg + CpG/A	1 month		VSR A	VSR B		[53]
	Adults aged 65–85 years						More than 10,000 and less than 100,000 GMT	More than 10,000 and less than 100,000 GMT		
Phase IIA	n = 70	2022	RSVpreF pre-fusion	120 μg	28 days		VSR A	VSR B		[54]
	Healthy adults 18–50 years of age						8795.7 GMT	8783.5 GMT		
Phase IIb	n = 327 (women) n = 403 (infants)	2022	RSVpreF pre-fusion	120 μg + Al(OH)_3_	On the day of delivery in newborns		VSR A	VSR B		[57]
	Healthy pregnant women 18–49 years of age and their infants						18,507 GMT	20,669 GMT		

GMT: geometric mean antibody titer; Alum: Aluminum adjuvant; GMT/C: geometric mean titer/antibody concentration; GMC: geometric mean concentration; AS01E/AS01B: AS01-based adjuvant; Al(OH)_3_: adjuvant aluminum hydroxide; CpG/A: oligodeoxynucleotide adjuvant CpG.

We compile data in Table 1 and Table 2 with the markers of immune response, such as anti-F IgG antibody titers, competitive antibody titers with Palivizumab, a monoclonal antibody known to protect infants against RSV, and neutralizing antibody titers. The data reported in most of the studies are on microneutralization antibody titers. In the studies that use the RSV vaccine based on post-F conformation, the results of microneutralization antibody titers range from 1000 to 2000 GMT in contrast to studies that used pre-F where the microneutralization titers range from 1000 to 100,000 GMT (Table 1 and Table 2). These data confirm that pre-F conformation is crucial to increase neutralization antibody levels.

Correlates of protection are used to assess whether and how levels of vaccine efficacy vary across vaccinated subgroups. Although there is not yet an established correlate of the protection of the RSV vaccine, data on palivizumab are important to guide an antibody threshold associated with protection. A study showed that maternal antibodies against the RSV F protein correlate with the protection of RSV causing lower respiratory disease in infants [66]. They evaluated the immunological results of the RSV F prefusion nanoparticle vaccine in mothers and their infants in South Africa who participated in phase III maternal immunization [66]. The next challenge is to understand whether this parameter can be applied as a correlate of protection to other RSV vaccine platforms in development. Also, the immunological results between the clinical trials are heterogenic, making finding a correlation of protection more complex.

#### 3.1.3. Synthetic Virus-like Particle (VLP)

The V-306 vaccine, funded by Virometix AG, is a subunit vaccine based on synthetic virus-like particles that contain multiple mimetics of proteins at the RSV F site, known as FsIIm. This vaccine was tested in a phase I, placebo-controlled study in healthy women aged 18 to 45 years, conducted in Belgium. The study assessed safety, reactogenicity, and immunogenicity. Participants who received the vaccine showed a significant increase compared to baseline levels in IgG antibody immune responses specific to the anti-FsIIm epitope. However, in terms of microneutralization titers, no significant changes were measured in RSV A microneutralization titers, and for RSV B, increases in titers were observed in six of the vaccine recipients and in two of the placebo recipients. Levels of anti-F IgG antibodies showed no significant changes after vaccine administration. The authors suggest several possible modifications to improve assay conditions and propose new ideas for designing a more effective vaccine, such as adding one or more antigenic epitopes to the vaccine to mimic additional neutralizing epitopes [67].

#### 3.1.4. Other RSV Proteins

The extracellular domain (SHe) of the small hydrophobic (SH) protein of HRSV was considered a proper antigen based on pre-clinical data [68]. In a phase I study, a synthetic peptide of 23 amino acids representing the ectodomain SH of RSV called RSV (A) was tested with two different adjuvants, regarding immunogenicity and safety. The DPX-RSV(A) vaccine combines the RSV(A) antigen with a synthetic lipopeptide adjuvant called DepoVax (DPX), which is a lipid mixture of cholesterol, phosphatidylcholine, and Montanide ISA VG, designed to enhance the vaccine’s immune potency by creating a unique local depot at the injection site The RSV(A)-Alum vaccine, on the other hand, combines the RSV(A) antigen with an aluminum hydroxide adjuvant [69,70]. The DPX-RSV(A) vaccines achieved geometric mean antibody titers 10 times higher on day 56 and almost 100 times higher on day 236 compared to the placebo. However, the immune responses developed by the RSV(A)-Alum vaccines were weak, showing no significant difference from the placebo group [69].

Another RSV protein studied as a vaccine target is the G protein. The G protein is responsible for viral binding, acting as a binding protein during RSV infection by interacting with target cell receptors in the host. The BARS13 vaccine is a subunit vaccine based on a recombinant G protein of RSV [71]. This vaccine contains two components: purified RSV-G, which functions as an antigenic component, and cyclosporine A (CsA), which acts as an immunomodulator and diluent to reconstitute RSV-G. CsA is an immunosuppressant widely used in organ transplants and autoimmune diseases because it can induce antigen-specific regulatory T cells to achieve tolerogenic responses when combined with a protein antigen. The vaccine was tested in a Phase I, placebo-controlled study in healthy adults aged 18 to 45 years, conducted in Australia. Higher doses and the two-dose regimen showed advantages in increasing antibody concentration [71].

### 3.2. Recombinant Vector Vaccines

This type of vaccine consists of benign genetically modified viral vectors, into which one or more antigenic genes from pathogens are inserted. These genes are expressed to ensure immunogenic activity [72]. There are several types of vectors, including viral vectors like adenoviruses (derived from chimpanzees [73,74,75,76,77] and serotype 26 [78,79,80,81,82], Modified Vaccinia Ankara virus [83,84,85,86,87], and Sendai virus [88], as well as bacterial vectors, including the use of BCG [89].

#### 3.2.1. Viral Vaccine Vectors

The PanAd3-RSV and MVA-RSV vaccines are developed, respectively, from chimpanzee-derived adenovirus vectors and Modified Vaccinia Ankara virus, genetically modified to express the F, N, and M2-1 proteins of RSV. A deletion of adenovector genes is made to prevent its replication, and MVA is unable to replicate in mammalian cells. In a phase I study involving healthy adults aged 19 to 48 (n = 42), the safety and immunogenicity of these two vaccines were evaluated when administered in four different prime-boost combinations, including intranasal administration. Geometric mean titers (GMTs) against RSV increased in response to the I.M. administration of PanAd3-RSV, peaking 4 weeks after both combined doses, representing a 1.7-fold increase. Following I.N. administration, GMTs remained at baseline levels 8 weeks after the first dose, only increasing 4 weeks after the I.M. booster (1.40 times with MVA-RSV and 1.39 times with PanAd3-RSV). The IFN-γ-producing T-cell response was evaluated after stimulation with four peptide sets (Fa, Fb, M, and N), and the magnitude was higher 2 weeks after I.M. initiation compared to I.N. The MVA-RSV vaccine generated an over 10-fold increase in RSV-specific T-cell responses compared to baseline levels, regardless of the route of the first dose. The vaccine was shown to be safe and immunogenic, suggesting further studies in target RSV populations [73]. In another phase I study, which was an expansion of the previous one, the two vaccines were tested in 30 healthy elderly individuals between 60 and 75 years old [74]. The objectives were to evaluate the safety and immunogenicity of the two vaccines in four different combinations, including a single-dose administration of MVA-RSV. Regarding safety, the main local adverse events of the IN vaccine were nasal discharge and irritation, and for the IM vaccines, the main local adverse event was local pain, while the systemic events were headache, malaise, myalgia, and arthralgia, with no adverse events related to the vaccination. The increase in the mean titers of neutralizing RSV serum antibodies in older adults was 2.2-fold on day 28 after PanAd3-RSV IM, and the boost did not cause further increases. In contrast, a single vaccination with MVA-RSV IM induced an average change of 2.4-fold. The IN route did not induce any significant change in serum antibody titers, only after the MVA-RSV IM boost, which was 1.71-fold. The responses of IFNγ-, IL2-, and IL4-producing CD4+ and CD8+ T cells were similar between younger and older adults. From these results, it was observed that PanAd3-RSV and MVA-RSV were safe, and compared to data from younger adults who received the same vaccines, there was practically no loss in the potency of boosting desirable immune responses for RSV [75].

Another vaccine developed with the Modified Vaccinia Ankara virus was tested in a phase I study, using the strain MVA-BN, which lacks replication capacity and encodes the surface proteins F and G of RSV subtypes A and B, as well as the internal N and M2 proteins [82]. The objectives were to evaluate its safety, reactogenicity, and immunogenicity in healthy adults aged 18 to 65 (n = 63). Two doses were administered intramuscularly, with a 4-week interval between them, at two different levels. The lower dose (1 × 10^7^ TCID50) was given only to adults aged 18 to 49, while the higher dose (1 × 10^8^ TCID50) was administered to participants in all age groups of the trial. Regarding safety and reactogenicity, the most frequent adverse events were pain, erythema, and itching at the injection site and, systemically, headache, fatigue, and elevated body temperature, though below grade 3. Concerning immunogenicity, the geometric mean spot-forming units (GMSFUs) of IFN-γ-secreting T cells increased approximately twofold compared to baseline in all vaccinated groups in response to peptides representing RSV’s F, G (A and B), M2, and N proteins, peaking one week after vaccination, with no significant difference after the second dose. The greatest response in the group that received the low dose was to M2 (62.5%), 93.8% of young adults who received the high dose responded to M2, and 88.9% responded to G(A); 72.2% of older adults who received the high dose responded to M2 and 94.4% to G(A). The GMSFUs specific to RSV in IL-4-producing cells were very low, indicating a Th1-biased response. Additionally, the vaccination induced an increase in RSV-specific antibody levels, and humoral responses were generally observed two weeks after the first vaccination, reaching peak values at variable times. A significant difference in neutralizing antibody GMFIs was only observed in the high-dose group (18–49), where increases of 1.6 times for PRNT A and 1.2 times for PRNT B were observed, while the other groups had GMFIs similar to the placebo. The low neutralization may suggest that the IgG response to RSV was not specifically directed toward the F protein. [82].

A phase II placebo-controlled study was conducted in adults aged 55 and older testing the MVA-BN vaccine (n = 420) [83]. Its objective was to identify the ideal dose and vaccination schedule, testing a low dose (1 × 10^8^ InfU—infectious units) or a high dose (5 × 10^8^ InfU) and two low doses or two high doses, administered intramuscularly with a 4-week interval between them, and a booster dose 12 months later. The booster doses administered did not show a significant impact on most immunological parameters, regardless of dose. The single high dose resulted in an equal or greater immune response compared to all other doses. The vaccine was considered safe, inducing an immune response that persisted for 6 months [83]. Next, a phase IIa placebo-controlled study was conducted in healthy adults aged 18 to 50 in London, UK, between January and November 2021 (n = 74) [84]. The study’s aims were to evaluate the safety, immunogenicity, and efficacy of a single dose of the RSV-A vaccine, at 5 × 10^8^ infectious units/0.5 mL, after challenge with RSV-A Memphis 37b at a dose of 4.5 log_10_ PFU. There were two serious adverse events of asymptomatic myocarditis, probably related to RSV-A inoculation. The GMTs of IgA and IgG levels increased 3.7 and 4 times, respectively, two weeks after MVA-BN-RSV vaccination, but there was no increase in response to the challenge. When infection was defined by viral load ≥ LLOQ (lower limit of quantification), efficacy ranged from 51.8% to 79.3%, and when defined solely by virus culture results, efficacy was 88.5%. The vaccine proved to be safe, immunogenic, and highly effective against infection in the adult population [84]. Phase III of the MVA-BN vaccine against RSV was performed, recruiting 18,348 adults aged ≥ 60 years; the vaccine did not meet the primary objective and its efficacy was 42.9 % against RSV-associated lower respiratory tract disease and 48.8 % against acute respiratory disease [85]. After 14 days of immunization MVA-BN-RSV induced a humoral immune response in terms of neutralizing antibodies. The results for antibodies against RSV A was 482 GMT, and against RSV B, it was 475.3 [85].

Replication-deficient viral vector adenovirus-155, derived from chimpanzees, into which DNA fragments encoding the RSV F, N, and M2-1 proteins were inserted, was also tested as a potential RSV vaccine (ChAd155-RSV). The first clinical trial of this vaccine was a phase I, placebo-controlled study, conducted between July 2015 and January 2016, with the objective of evaluating the safety, reactogenicity, and immunogenicity of the vaccine by administering two intramuscular doses at two levels and with a 1-month interval between them [75]. The participants were healthy adults aged 18 to 45 (n = 72). There were no serious adverse events related to vaccination. In terms of immunogenicity, the average frequency of RSV-F-specific IFN-γ-secreting T cells was 2.69 times higher than baseline on day 7 and 3.09 times on day 30 after the first dose in volunteers who received the higher dose (5 × 10^10^ vp), with no increase after the second dose or in volunteers who received the lower dose (5 × 10^9^ vp), and similar trends were observed for RSV-N- and RSV-M2-1-specific cells. Seven days after the first dose of 5 × 10^10^ vp, the frequency of IgG-producing B cells specific for RSV-F was 133.3 per million PBMCs, and seven days after the second dose, it was 1, while that of IgA-producing cells specific for RSV-F was 16.7, with no difference after the second dose. An increase in the GMTs of neutralizing antibodies against RSV-A of 2.4 times was observed in the lower-dose group (to 1474) and 2.6 times in the higher-dose group (to 1293) 30 days after the first dose. No additional increase was observed with the second dose. It is believed that the decreased response after the second dose occurred due to pre-existing immunity against RSV and because some volunteers had antibodies that cross-reacted with the viral vector, reducing the induction of neutralizing antibodies (anti-RSV NAb). The results demonstrate that the vaccine increases immunogenicity without raising significant safety concerns in adults previously exposed to RSV [75].

In 2022, the ChAd155-RSV vaccine was evaluated in a phase I/II trial, conducted across 29 sites in Canada, Italy, Mexico, Panama, Spain, Taiwan, and the United States, involving RSV-seropositive children aged 12 to 36 months (n = 82). The objectives were to evaluate the safety, reactogenicity, and immunogenicity of two doses of the vaccine, administered intramuscularly at three dose levels (0.5 × 10^10^, 1.5 × 10^10^, and 5 × 10^10^ vp). No serious adverse events were related to vaccination. In terms of immunogenicity, by day 31 after the first dose, neutralizing antibody titers against RSV-A2 reached 771.7 GMT in the lowest-dose group, 1646 in the medium-dose group, and 2204 in the highest-dose group, with no additional increase after the second dose. On day 366, GMTs were 1.5 to 2 times higher in the medium- and high-dose groups compared to the placebo groups, and similar trends were observed for anti-RSV-F antibody concentrations. Based on these results, it was possible to conclude that the administration of the vaccine to RSV-seropositive children was well tolerated and had an acceptable safety profile up to two years, suggesting further studies in RSV-seronegative children are needed [76]. This ChAd155-RSV vaccine was also tested in infants 6 to 7 months old in a phase I-II trial, in which 149 were previously seronegative. The vaccine showed a safe profile, and in infants that were infected with RSV after vaccination, there was no evidence of vaccine-enhanced respiratory disease. At the end of the RSV season, the neutralization antibodies levels were 225 GMT in the group that received two high vaccine doses. The authors concluded based on the results that the target efficacy profile was unlikely to be met and the development of ChAd155-RSV was discontinued [77].

Also, based on the adenovirus vector, the Ad26.RSV.preF vaccine was designed using replication-deficient adenovirus serotype 26, carrying a DNA transgene expressing the F protein of the RSV strain A2 in its prefusion conformation. In a phase I placebo-controlled clinical trial, the safety and immunogenicity of one or two doses administered intramuscularly in healthy adults aged ≥ 60 years (n = 73) were evaluated at two dose levels, with one year between them [78]. Regarding safety, no grade 3 local adverse effects were reported. The higher dose was more immunogenic. These antibody titers decreased by day 186 but remained approximately 2 times higher than the placebo (1.5–2× for the low dose and 2–2.5× for the high dose) until day 365 when the second dose was administered. A similar trend was observed for pre-F specific antibody titers, with a proportional increase in RSV-B neutralization titers compared to RSV-A2. After 2 years, both remained above baseline and higher than the placebo. Additionally, the frequency of F-specific IFN-γ-secreting T cells increased 2.1 times or more compared to the baseline 28 days after the first dose and 2.3 times or more 28 days after the second dose at both dose levels, remaining above the baseline up to 2 years post vaccination. The vaccine was well tolerated, showed an acceptable safety profile, and was immunogenic after a single dose [78].

The safety and immunogenicity of the Ad26.RSV.preF vaccine was evaluated in a phase IIa trial, co-administering with a seasonal influenza vaccine (Fluarix Quadrivalent). The target population was the same age group (≥60 years) (n = 180), but participants could not have been vaccinated against seasonal flu during the 2017–2018 season [79]. Both vaccines are administered intramuscularly, either together or separately (one on day 1 and the other on day 29). No severe adverse events were associated with the vaccines. Pre-F binding antibodies increased 2.34-fold (ELISA units/L) in the co-administration group and 2.58-fold in the RSV vaccine alone group. Based on these results, it was concluded that co-administration of both vaccines is safe and does not interfere with immune responses [79]. The same group conducted another phase IIa, placebo-controlled clinical trial, using for the first time the established human viral challenge model to evaluate a candidate vaccine against RSV in healthy adults aged 18 to 50 years (n = 63) [80]. The study was conducted from August 2017 to November 2018 and assessed the safety, immunogenicity, and efficacy of a vaccine administered intramuscularly, with RSV-A inoculation administered intranasally (4 log_10_ PFU/mL) 25–33 days after immunization. The vaccine induced a 6.8-fold increase in the GMT of anti-pre-F IgG antibodies (ELISA units/L) from day 0 to 28 days post immunization, and from pre-challenge to 28 days post challenge, an 8.2-fold increase was seen in the vaccinated group and a 2.1-fold increase in the placebo group. The vaccine was well tolerated and has the potential to reduce viral transmission, suggesting further studies in at-risk populations [80].

The Ad26.RSV.preF vaccine was used for a phase I/IIa, placebo-controlled clinical trial in 12 adults aged 18 to 50 years and 36 children aged 12 to 24 months who were seropositive for RSV. It was conducted in the United States, Finland, and the United Kingdom, with the administration of two intramuscular doses 29 days apart. The pediatric dose was 5 × 10^10^ vp and the adult dose was 1 × 10^11^ vp, assessing safety, tolerability, and immunogenicity [81]. In adults, an increase in the titers of neutralizing antibodies against RSV A2, pre-F, and post-F binding proteins was observed 28 days after the first dose, and after the second dose, the titers remained stable for 28 days, decreasing modestly at 6 months. In children, the GMTs of neutralizing antibodies against RSV A2 increased 13.2 times on day 29 after the first dose and 18.47 times on day 57 compared to the baseline. The GMTs of pre-F and post-F specific antibodies increased 19.89 and 8.90 times, respectively, on day 29, and by day 57, there was a further increase of 32.5 times (pre-F) and 11.20 times (post-F) compared to the baseline, all remaining above the initial levels by the end of the study. Additionally, on day 29, the median values of IFN-γ-, TNF-α-, or IL-2-secreting CD4+ T cells increased 3-fold, and CD8+ T cells increased 2-fold, showing a response profile biased towards Th1 through the increase in CD4+ IFN-γ-secreting T cells. The vaccination also induced cross-neutralizing antibodies against the RSV-B strain in children, with GMTs increasing 27.9 times on day 57. Due to the occurrence of transient fevers commonly observed in children and adults, it was suggested that there should be a reduction in the vaccine in seronegative children. These results demonstrate that Ad26.RSV.preF is immunogenic for healthy adults and children, with no safety concerns raised [81].

Lastly, the Ad26.RSV.preF was administered intramuscularly in a phase IIb placebo-controlled study conducted in 40 centers in the United States. The safety, immunogenicity, and efficacy of the combined vaccine were evaluated in adults aged ≥ 65 years (n = 5782). No serious or fatal adverse events related to vaccination were reported [82]. The GMT of neutralizing antibodies against RSV A2 increased 12.1 times from baseline up to day 15 and remained 5.5 times higher by day 169. Similar results were seen for RSV B, with a 9.4-fold increase by day 15 and a 4.4-fold increase by day 169. The average frequency of RSV-F-specific IFN-γ-secreting T cells increased 13-fold (SFCs/10⁶ PBMCs) by day 15, remaining above baseline for at least 6 months. The vaccine’s efficacy in the study’s global population was 80.0% for more severe disease and 69.8% for any acute respiratory infection caused by RSV. Given these results, it was observed that the Ad26.RSV.preF vaccine combined with the pre-F protein is safe, immunogenic, and effective against lower respiratory tract diseases caused by RSV in adults aged 65 years and older, even in the presence of additional risk factors for severe RSV disease [82]. The long-term efficacy of this vaccine was published in 2024. Vaccine efficacy against RSV lower respiratory tract disease was 76·1% over seasons 2 and 3 and 78·7% across three seasons. The neutralizing antibodies against RSV A2 increased by 12 times from baseline to day 15 post vaccination and remained 2.8 high at day 365 [83].

The Sendai virus, a strain of Parainfluenza Virus type I, was also investigated as a vector to develop an RSV vaccine. The gene of the F protein isolated from RSV-A2 was inserted into a Sendai replication-competent vector (SeVRSV). This vaccine was tested in a phase I placebo-controlled study in a single dose of the vaccine (1 × 10^7^ EID50) administered intranasally to healthy adults aged 18 to 45 years (n = 21). No serious adverse reactions occurred. Regarding immunogenicity, IgG antibody levels specific to the RSV F protein increased by 1-fold in GMTs from day 0 to day 28 post vaccination (GMFR—geometric mean fold rise of 1.1). This study showed that although SeVRSV was well tolerated, there was minimal antibody response to SeV and an insignificant response to the RSV F protein in these RSV-seropositive adults [88].

#### 3.2.2. Bacterial Vector

The rBCG-N-RSV vaccine uses the recombinant Calmette–Guérin bacillus (BCG) vector from *Mycobacterium bovis*, which expresses the RSV nucleoprotein. This vaccine was evaluated in a phase I clinical trial conducted in Chile, using as control the standard BCG. The aims were to evaluate the safety, tolerability, and immunogenicity of a single dose of the vaccine administered intradermally to healthy men aged 18 to 50 years, with dose escalation across three levels (n = 24) [89]. Starting from 14 days post vaccination, there was an increased capacity for RSV neutralization, especially in volunteers who received the lowest dose, likely because those vaccinated with other doses may have recently been exposed to seasonal infections. Additionally, there was an increase over time in all vaccinated participants, regardless of the dose, with practically no difference in the control group. There was an increase in IFN-γ-secreting cells in response to RSV-N in individuals who received medium doses on day 60 post vaccination. An increase in IL-2-secreting cells was also observed in volunteers who received the high dose, from day 14 to day 30 post vaccination. However, IL-2 levels returned to baseline between days 60 and 180 of monitoring [89].

### 3.3. Live Attenuated Virus Vaccines

Live attenuated virus vaccines contain the live version of the causative virus, weakened in the laboratory through genetic modifications. This type of vaccine is known to elicit a strong immune response, providing immunity for an extended period in the vaccinated individual’s life. Efforts to develop live attenuated RSV vaccines were initiated following the tragic outcomes of studies involving a formalin-inactivated pediatric RSV vaccine in the 1960s, where the vaccine offered poor protection and, in RSV-naïve recipients, primed them for enhanced disease upon subsequent natural RSV infection. Live attenuated vaccines were considered safe for individuals who were not previously exposed to RSV, also known as RSV-naïve recipients, and not one of the vaccinees showed evidence of enhanced disease after natural infection with wild-type RSV [90].

Live attenuated RSV vaccines are administered intranasally, providing three key advantages: (1) they replicate in the upper respiratory tract and remain immunogenic even in the presence of maternally derived neutralizing antibodies, which are typically present in young infants; (2) they induce local mucosal immunity, crucial for limiting the replication of respiratory viruses; and (3) they allow for needle-free administration. This vaccine type is one of the primary options for children over six months, given its safety in recipients who have never been exposed to the virus [91,92].

#### 3.3.1. Medi-559 Vaccine

The MEDI-559 vaccine (administered intranasally) pioneered the live attenuated virus vaccine type for RSV, serving as the foundation for subsequent vaccines of the same type that were tested sequentially. MEDI-559 was tested at 28 sites in the United States in infants and children aged 5 to 23 months. A phase I study on it, a placebo-controlled type, was published in 2013 and it assessed the safety and immunogenicity of the vaccine. Regarding its adverse effects, one vaccine recipient experienced Bell’s palsy 210 days after the administration of the second dose without complications or sequelae. The event was not associated with the vaccine due to the latency period. Another vaccine recipient developed bronchiolitis 2 days after the second dose, considered a possible serious adverse event. The study found that 95% of individuals developed a fourfold increase in at least one measure of humoral immunity (specific IgG for F, IgA, or RSV microneutralization) after receiving three doses of the vaccine. This study demonstrated that the vaccine is biologically active and immunogenic in the pediatric population, requiring further studies to determine its safety [92].

#### 3.3.2. Medi ΔM2-2 Vaccine

MEDI ΔM2-2 is an intranasal live attenuated virus vaccine. It lacks the M2-2 gene, which positively regulates viral gene expression and leads to virus attenuation. The RSV MEDI ΔM2-2 vaccine was tested in a phase I study in adults and RSV-seropositive and -seronegative children. The study, conducted in the United States, was an open-label clinical trial in adults and a double-blind, placebo-controlled randomized clinical trial in the pediatric population. The vaccine was well tolerated, with limited observed adverse effects and no evidence of increased RSV disease. Vaccine replication was highly restricted in RSV-seronegative children. Some vaccinated individuals experienced substantial increases in RSV-specific serum-neutralizing antibody titers without reported disease, suggesting protection against significant disease after exposure to wild-type RSV. The study also demonstrated the stability of the ΔM2-2 vaccine virus attenuation phenotype, alleviating concerns about potential transmission. With its highly restricted replication, increased immunogenicity, and stable attenuation phenotype, the MEDI ΔM2-2 vaccine appears promising as a candidate for the development of a live attenuated RSV vaccine. Findings suggest its potential to induce protective immune responses without causing an increase in disease, addressing a critical need for an effective RSV vaccine [93].

#### 3.3.3. RSVΔG Vaccine

The RSVΔG vaccine (administered intranasally) is a live attenuated virus vaccine that uses the non-sterile recombinant RSV strain from serogroup A, lacking the G binding protein [94]. In a placebo-controlled phase I study conducted in the Netherlands, the vaccine was tested in healthy non-smoking adults aged 18 to 50. The test aimed to evaluate the vaccine’s safety and tolerability. Additionally, it assessed the viral load of the vaccine and the virus excretion in vaccinated individuals, as well as its immunogenicity. The vaccine was well tolerated by all participants, with local symptoms such as sneezing and runny nose being the most reported. Concerning the viral load after vaccine administration, specific RSV RNA was found in 8.3% of individuals who received the vaccine. The study suggests that the lack of correlation between the increase in IgA titers and other immunogenicity parameters or viral clearance may be due to the limited viral replication of the vaccine in healthy adults with pre-existing neutralizing antibodies. It is also possible that the 6.5 log_10_ dose was insufficient to overcome natural immunity and induce an immune response in adults. Additionally, the nasal lavage approach used in the study may have underestimated mucosal immune response. Therefore, the tested dose showed no clear signs of inducing an immune response in seropositive adults, suggesting that RSVΔG should undergo future dose escalation tests in adults [94].

The study group “The International Maternal Pediatric Adolescent AIDS Clinical Trials (IMPAACT),” with primary funding from the “National Institute of Allergy and Infectious Diseases of the National Institutes of Health,” tested different live attenuated virus vaccines, all derived from the cDNA of the RSV subgroup A strain A2. Among them are RSVcps2 [95], LIDΔM2-2 [96], LID/ΔM2-2/1030s [97], RSV/ΔNS2/Δ1313/I1314L [98], D46/NS2/N/ΔM2-2-HindIII [99] and RSV/276 [100]. RSVcps2 is a vaccine carrying the stabilized 248 mutations (248s) and the 1030s mutation. It has three main changes compared to its precursor, the MEDI-559 vaccine. These three major changes in the gene encoding M2-1 and M2-2 proteins are as follows: in the stabilized 248 mutation, it contains 831L (TTG); in the 1030 mutation, asparagine was modified to lysine [1321K(AAA)]; and the codon near 1313S(AGC) was changed to 1313S(TCA) [95]. The LIDΔM2-2 vaccine uses the cDNA-derived version of the RSV subgroup A strain A2 with a deletion of the RNA synthesis regulation M2-2 protein. Changes were made to the M2-2 ORF and SH gene to stabilize RSV full-length cDNA plasmids during bacterial propagation [96]. The LID/ΔM2-2/1030s vaccine is derived from the LIDΔM2-2 vaccine, and in this new version, a “1030s” mutation was added to the L polymerase protein, consisting of a temperature-sensitive attenuation mutation during vaccine propagation in bacteria [97]. The RSV/ΔNS2/Δ1313/I1314L vaccine contains two independent attenuating elements to improve cDNA stabilization during bacterial propagation [98]. The D46/NS2/N/ΔM2-2-HindIII vaccine uses the LIDΔM2-2 vaccine structure but has a deletion of 234 nucleotides in M2-2 [99]. Lastly, the RSV/276 vaccine has a deletion of 234 nts in the M2-2 ORF. RSV/276 differs from recombinant RSV A2 (KT992094) by 19 nt across the genome, including two coding changes (K51R in ORF NS2; T24A in ORF NO) [100]. The RSV/6120/ΔNS2/1030s vaccine was evaluated in a phase I, double-blind, placebo-controlled trial in seronegative children. This vaccine is identical to RSV/ΔNS2/Δ1313/I1314L except that the Δ1313/I1314L mutations in the L polymerase were replaced by the 1030s missense mutations S1313(TCA) and Y1321K(AAA) to become more immunogenic and less restricted in replication. This vaccine was immunogenic, but the frequency of rhinorrhea in vaccines was higher than in the placebo group [101]. More information about the immune response induced by the vaccines tested by the IMPAACT group is contained in Table 3. The data of RSV neutralization antibodies from the studies demonstrated in Table 3 are expressed differently than the studies in Table 1 and Table 2, which are hard to compare. However, it suggested that the pre-fusion-based vaccines induced higher levels of neutralizing antibodies than live attenuated vaccines.

**Table 3:** Results of the IMPAACT study group regarding tests of different live attenuated vaccines, all derived from the cDNA of the RSV subgroup A strain A2.

Vaccine	Recipents, No	Shed Vaccine Virus, No (%) *b	Serum RSV Neutralizing Ab Titer *c				Serum Anti-RSV F IgG Ab Titer *e			
			Vaccination		RSV Surveillance Season		Vaccination		RSV Surveillance Season	
			Before	After *d	Before	After	Before	After *d	Before	After
RSVcps2	Vaccine (n = 34)	29 (77%)	2.3 (2.3–4.2)	5.3 (4.1–6.2)	5.1 (4.0–6.1)	7.2 (5.6–8.8)	5.6 (4.6–9.6)	11.6 (9.6–11.6)	11.6 (7.6–11.6)	13.6 (11.6–15.6)
	Placebo (n = 16)	0	2.9 (2.3–4.9)	2.3 (2.3–5.6)	2.3 (2.3–5.2)	6.1 (3.5–8.1)	7.6 (4.6–10.6)	5.6 (4.6–11.6)	5.1 (4.6–11.6)	11.6 (7.1–14.6)
LIDΔM2-2	Vaccine (n = 20)	19 (95%)	2.3 (2.3, 3.4)	7.3 (6.6, 8.5)		7.3 (6.7, 9.3)	5.6 (4.6–7.6)	11.6 (10.6–13.6)		11.6 (10.6–13.6)
	Placebo (n = 9)	0	2.3 (2.3, 3.9)	2.3 (2.3, 2.3)		2.3 (2.3, 6.0)	9.6 (7.6, 9.6)	7.6 (5.6, 9.6)		9.6 (5.6, 11.6)
LID/ΔM2-2/1030s	Vaccine (n = 20)	15 (85%)	2.3 (2.3–2.3)	6.4 (5.7–7.1)	6.2 (5.6–7.1)	6.6 (5.6–8.8)	7.1 (5.9–8.9)	14.0 (12.7–14.8)	13.8 (12.7–14.8)	13.7 (12.6–15.6)
	Placebo (n = 11)	0	2.9 (2.3–2.3)	2.3 (2.3, 2.3)	2.3 (2.3–2.3)	6.2 (5.6–7.9)	7.2 (5.9–8.8)	6.7 (4.6–8.9)	6.8 (5.2–8.9)	15.2 (14.8–15.6)
RSV/ΔNS2/Δ1313/I1314L *a	Vaccine dose 5 (n = 15)	11 (73%)	2.9 (1.0) *	5.2 (1.7) *	5.2 (1.6) *	6.9 (2.4) *	8.0 (2.9) *	11.5 (2.2) *		
	Placebo (n = 7)	0	2.8 (0.8) *	2.7 (0.9) *	2.7 (0.9) *	4.9 (2.4) *	6.1 (2.3) *	6.2 (2.3) *		
	Vaccine dose 6 (n = 20)	18 (90%)	2.4 (0.6) *	6.0 (1.9) *	5.6 (1.5) *	7.1 (2.9) *	7.2 (2.3) *	13.0 (2.4) *		
	Placebo (n = 10)	0	2.3 (0.0) *	2.4 (0.4) *	2.4 (0.4) *	5.3 (1.7) *	6.4 (1.6) *	5.6 (1.4) *		
D46/NS2/N/ΔM2-2-HindIII	Vaccine (n = 21)	20 (95%)	2.3 (2.3–2.3)	7.2 (6.1–8.3)	6.5 (5.2–8.4)	6.4 (5.8–10.3)	9.4 (7.3–10.2)	15.7 (13.9–15.7)	13.9 (12.5–15.7)	13.9 (13.0–15.7)
	Placebo (n = 11)	0	2.3 (2.3–2.3)	2.3 (2.3–2.3)	2.3 (2.3–2.3)	2.3 (2.3–6.0)	8.5 (7.2–10.1)	8.1 (6.0–9.1)	7.6 (6.0–8.5)	8.6 (6.2–15.7)
RSV/276	Vaccine (n = 24)	22 (92%)	2.3 (2.3–2.3)	6.7 (6.0–7.8)	6.7 (6.1–8.3)	6.4 (5.8–7.5)	7.2 (6.1–8.6)	12.6 (11.3–13.3)	12.3 (11.3–12.6)	12.3 (11.5–12.9)
	Placebo (n = 12)	0	2.3 (2.3–2.9)	2.3 (2.3, 2.3)	2.3 (2.3–2.3)	2.3 (2.3–5.8)	6.8 (5.4–9.5)	6.4 (4.6–7.9)	4.6 (4.6–6.5)	4.6 (4.6–11.9)
RSV/6120/ΔNS2/1030s soropositive young chlidren	Vaccine (n = 10)	2 (20%)	7.4 (0.7)	7.1 (1.0)	ND	ND	12.2 (1.5)	12.1 (1.5)	ND	ND
	Placebo (n = 5)	0	7.4 (0.6)	7.2 (0.7)	ND	ND	13.1 (0.8)	13.1 (0.8)	ND	ND
RSV/6120/ΔNS2/1030s soronegative young chlidren	Vaccine (n = 20)	20 (100%)	2.8 (0.9)	6.5 (1.1)	6.5 (1.2)	7.8 (2.7)	6.4 (2.0)	10.9 (1.7)	ND	ND
	Placebo (n = 10)	0	2.9 (0.9)	2.6 (0.6)	2.6 (0.6)	5.6 (3.2)	6.7 (1.6)	6.0 (2.0)	ND	ND

Titer results are expressed as median reciprocal log2 values (with IQRs), determined for all participants in each group. Specimens with titers below the limit of detection were assigned reciprocal titers of 2.3 log2 (PRNT60) and 4.6 log2 (ELISA). *a = doses are in Log_10_ PFU/mL; *b = defined as the detection of vaccine virus shedding in NW specimens by culture and/or by real-time qPCR during the first 28 days after inoculation; *c = PRNT as the analysis method; *d = at day 56 of the study; *e = ELISA as the analysis method; * = standard deviation (SD).

### 3.4. mRNA Vaccine

In this innovative vaccine model, instead of injecting the antigen into the body, it uses messenger RNA to instruct cells to produce specific antigens, thereby triggering a protective immune response against various infectious diseases [102,103]. These antigens are subsequently displayed on the cell surface, allowing the immune system to recognize it. This process promotes both cellular and humoral immunity, enhancing T cell-mediated responses and the production of neutralizing antibodies. A key advantage of this technology is its rapid antigen design and scalable production, eliminating several steps of production and purification [104,105].

#### 3.4.1. mRNA-1777 (V171)

An mRNA vaccine to protect against RSV infection has been developed, encoding the sequence of the RSV F protein in its pre-fusion conformation. This vaccine was tested in a phase I, placebo-controlled study in young adults aged 18 to 49 and healthy older adults aged 60 to 79, conducted in Australia. Safety, tolerability, and immunogenicity were evaluated [106]. The most reported adverse events were local injection pain and tenderness, with similar numbers of participants reporting these in the treatment groups. The most reported systemic adverse events in the treatment group included fatigue, generalized myalgia, headache, and malaise. These events were reported in higher numbers by participants receiving the 300 μg treatment dose. Regarding neutralizing antibodies, younger adults on day 29 post vaccination with the 200 μg dose showed geometric mean titers (GMT) of 1358.5 for RSV A and 965.1 for RSV B. Older adults showed higher antibody levels with the 300 μg dose, presenting geometric mean titers of 1719.9 for RSV A and 1424.0 for RSV B. The 200 μg dose presented the highest serum antibody titers for the pre-fusion F protein in both older and younger adults, with geometric mean titers ranging between 1,000,000 and 1,500,000 in younger adults and values above 1,500,000 in older adults. The participants’ serum antibodies were also assessed for their ability to compete with palivizumab or the D25 antibody. The D25 antibody specifically recognizes an epitope at site Ø, which is present only in the pre-fusion conformation of the F protein of the virus. The GMFIs and GMCs (geometric mean concentration) of the competing antibodies against palivizumab increased by 1.8 times for the 100 μg group on day 29 in younger adults, and in older adults, there was also an increase of 1.8 times for the 100 μg group. The competing antibody D25 increased by 3.7 times in the 100 and 200 μg groups from day 1 to day 29 in younger adults, while in older adults, there was 4.1 times increase for the 300 μg group over the same period. These data support further studies [106].

#### 3.4.2. mRNA-1345

The mRNA-1345 vaccine is an mRNA-based vaccine encapsulated in lipid nanoparticles, encoding the F glycoprotein of RSV. This glycoprotein is derived from the RSV A strain and stabilized in the virus’s pre-fusion conformation [107,108]. This vaccine was initially evaluated in a phase I, placebo-controlled study conducted in healthy adults aged 65 to 79 in the United States. The safety, reactogenicity, and immunogenicity of a single injection and a booster at different dose levels were assessed. All doses were well tolerated. Regarding immunogenicity, the highest dose of 200 μg showed 31,084.4 GMT of neutralizing antibodies against RSV A and 18,183.8 GMT against RSV B. These values remained above baseline for 12 months after a single injection. Following a booster injection 12 months after the initial dose, there was an approximately twofold increase in antibody titers, although numerically lower than those achieved after the first vaccination. These data support the continued development of this vaccine [107].

The next study of this vaccine was a phase II/III study, a placebo-controlled trial of a single 50 μg dose in adults aged 60 years and older, conducted across 22 countries. The primary objectives were to evaluate the efficacy of a single dose of the vaccine in preventing a first episode of RSV-associated lower respiratory tract disease with at least two lower respiratory symptoms and with at least three lower respiratory symptoms within 14 days to 12 months after vaccination. A secondary objective was to assess the efficacy of a single dose in preventing the first episode of RSV-associated acute respiratory illness with at least one symptom within 14 days to 12 months after vaccination. Another secondary objective was to evaluate the vaccine’s efficacy in preventing the first episode of RSV-associated lower respiratory tract disease according to the virus subtype. A single dose of the vaccine showed no evident safety concerns. Regarding immunogenicity, the primary efficacy analysis against RSV-associated lower respiratory tract disease with at least two signs or symptoms showed an efficacy of 83.7%. For RSV-associated lower respiratory tract disease with at least three signs or symptoms, the vaccine achieved an efficacy of 82.4%. For the secondary endpoints of RSV-associated acute respiratory illness, the vaccine efficacy was 68.4%. Concerning viral subtypes, the vaccine’s efficacy against RSV-associated lower respiratory tract disease was 91.7% and 90.0% for RSV A and 68.5% and 71.5% for RSV B [108]. This study demonstrated that a single dose of this vaccine was effective against a spectrum of confirmed RSV respiratory illnesses and was subsequently confirmed through the publication of the immunogenicity results from this same study, showing neutralizing antibody values of 21,475.4 GMT on day 29 for RSV A and 7246.0 GMT for RSV B, as well as 81,884.2 GMC for F protein-binding antibodies 29 days after vaccination. [108,109]. This vaccine was approved on 31 May 2024, for use in adults aged 60 and older under the brand name mRESVIA by the United States Food and Drug Administration (FDA) [110].

## 4. Conclusions

The increase in the neutralization antibody titers due to the pre-fusion conformation of F proteins used in RSV vaccines led to the approval of the vaccines. Two different vaccine types were approved: subunit and mRNA vaccines. Other RSV vaccine types have been tested, including recombinant vector vaccines and live attenuated virus, currently in phase III clinical trials.

Approved vaccines for adults have shown efficacy throughout one RSV season, but recent studies suggested a longer protection. For newborns, vaccines administered to pregnant women provided significant protection in the first 6 months of life, a critical period for RSV-related lower respiratory infections and hospitalizations. Given the high burden of RSV in young children, there is a need for further vaccine development to protect children beyond 6 months of age. The live attenuated vaccine type is one of the primary options for children over six months, given its safety in recipients who have never been exposed to the virus.

## Data Availability

No new data were created or analyzed in this study. Data sharing is not applicable to this article.
